# A pH-Triggered TELSAM Approach for Rapid, Tag-Free Protein Purification and Crystallization

**DOI:** 10.1063/4.0001137

**Published:** 2025-10-27

**Authors:** Wisdom Oshireku Abiodun, Celeste Litchfield, Mikaela Burtch, Jamison Cartwright, James D Moody

**Affiliations:** Brigham Young University, Provo, Utah, USA

## Abstract

Protein purification is a major and indispensable step in structural biology as it directly impacts the quality and success of structural studies. Requirements of high purity, sample homogeneity, protein stability preservation, and ultimately, crystallization facilitation are crucial in protein structure determination using X- ray crystallography. Unfortunately, the process from protein expression to purification is time and cost intensive. Thus, the need for a crystallization method that is reliable, time efficient, and requires reduced resources. Translocation ETS Leukemia (TEL) protein sterile alpha motif (SAM) (TELSAM) is a pH-triggered crystallization chaperone that facilitates protein crystallization by forming a helical polymer which helps to pre-organize copies of the target protein, making it easier for crystals to form. At high pH, TELSAM fusions are soluble and easy to purify and at low pH, TELSAM fusion forms a polymer that includes the permanently attached protein of interest. Cycles of pH drop, polymerization, and precipitation of TELSAM fusions alternating with cycles of pH increase, depolymerization, and resolubilization allow TELSAM fusions to be quickly separated from impurities or concentrated into small volumes. With this property of TELSAM, we can eliminate affinity tags and concentrators thereby significantly reducing purification time and cost. Using the appropriate pH buffering system, we tested this property of TELSAM fusions against multiple protein constructs and have gotten diffraction quality crystals.

